# Percutaneous Nephrostomy and Ureteroscopic Management of Bilateral Renal Papillary Necrosis in an Elderly Female

**DOI:** 10.7759/cureus.97831

**Published:** 2025-11-26

**Authors:** Islam Ibrahim Elwerdany Abosalem, Abdulaziz Saleh Alanazi, Ahmed Abu El Gasim Abdelrahman Dafaalla, Omar Adel Albsheish, Rakan Jazi Almokhlef, Munir Abdulhamid M Almayouf, Syed Rafiuddin Shah

**Affiliations:** 1 Department of Urology, King AbdulAziz Specialist Hospital - Sakakah, Sakakah, SAU

**Keywords:** adverse effects, anticoagulants, endourology, obstructive uropathy, percutaneous nephrostomy, renal papillary necrosis, ureteroscopy

## Abstract

Renal papillary necrosis (RPN) is a rare but clinically significant cause of obstructive uropathy, often associated with infection, diabetes mellitus, analgesic abuse, or systemic vascular comorbidities. Timely diagnosis and intervention are essential to prevent irreversible renal injury and sepsis. An 84-year-old female with multiple chronic comorbidities, including hypertension, ischemic heart disease, and long-term anticoagulation therapy, presented with acute severe left-sided loin pain, fever, and vomiting. Imaging revealed bilateral hyperdense lesions at the ureteropelvic junctions, initially mimicking calculi. Flexible ureteroscopy later confirmed sloughed necrotic papillae, consistent with bilateral RPN. The patient underwent urgent percutaneous nephrostomy for decompression, followed by definitive ureteroscopic retrieval of necrotic tissue. Histopathology demonstrated fibrosis, tubular epithelial erosion, and ischemic necrosis without evidence of malignancy. The patient showed marked clinical improvement, normalization of renal function, and complete radiological resolution at one-month follow-up. This case underscores the importance of early recognition, prompt decompression, and coordinated multidisciplinary management in obstructive RPN. A structured diagnostic and therapeutic approach can lead to full functional recovery even in elderly patients with complex comorbidities.

## Introduction

Renal papillary necrosis (RPN) is a rare but clinically important cause of obstructive uropathy, characterized by ischemic necrosis of the renal papillae and inner medulla. It is frequently linked to underlying conditions such as diabetes mellitus, urinary tract infections, analgesic abuse, and urinary tract obstruction [1,2]. The underlying pathophysiology involves compromised medullary blood flow, leading to tissue ischemia, necrosis, and eventual sloughing of papillae - an event that can precipitate acute obstruction and rapid clinical deterioration [3]. Although considered uncommon, RPN often presents with nonspecific symptoms and may closely mimic other etiologies of obstruction, which contributes to delays in diagnosis and management [4].

This case report describes an 84-year-old female with multiple comorbidities who developed bilateral RPN, manifesting with acute left loin pain, fever, and vomiting. Prompt intervention with percutaneous nephrostomy (PCN), followed by ureteroscopic identification and extraction of sloughed papillae, resulted in full clinical recovery. The successful outcome highlights the value of early decompression and definitive endoscopic clearance in preventing progression to sepsis or renal failure. Most importantly, this case reinforces the need for clinicians to consider RPN in elderly patients presenting with features of obstructive uropathy - particularly when risk factors such as diabetes, recurrent infections, anticoagulant use, or vascular compromise are present. Early recognition and timely intervention can significantly improve patient outcomes and reduce morbidity associated with this under-recognized condition.

Objective

To highlight the clinical presentation, diagnostic challenges, and effective management strategies for bilateral RPN in an elderly patient with multiple comorbidities.

## Case presentation

Initial evaluation

An 84-year-old female with multiple chronic comorbidities, including hypertension, ischemic heart disease, and long-term anticoagulation therapy for atrial fibrillation, presented to the emergency department with acute, severe left-sided colicky loin pain for two days, radiating anteriorly. The pain was associated with high-grade fever with rigors, multiple episodes of non-bilious vomiting, and subjective oliguria during the same period. She denied dysuria, hematuria, or other lower urinary tract symptoms.

On examination, she appeared ill and febrile (38.9°C), with tachycardia and mild hypotension. Marked tenderness and guarding were elicited over the left renal angle. Her medical history was notable for recurrent urinary tract infections, though she denied diabetes mellitus or chronic analgesic use.

Initial laboratory investigations revealed leukocytosis (total leukocyte count = 15,200/µL) with neutrophilic predominance, and elevated serum creatinine (185 µmol/L; baseline ≈ 88 µmol/L), suggestive of acute kidney injury (AKI). C-reactive protein was markedly raised, indicating a severe inflammatory or infectious process. Urinalysis demonstrated pyuria and proteinuria without significant hematuria. Blood cultures were obtained before starting broad-spectrum intravenous antibiotics. The trend of her hematologic and biochemical parameters throughout management is summarized in Table 1.

**Table 1:** Clinical, laboratory, and imaging findings in the patient with bilateral renal papillary necrosis. KUB: kidneys, ureters, and bladder; DJ stent: double-J stent

Parameter	Findings	Additional Details
Age/Sex	An 84-year-old female	Frail, multiple comorbidities (hypertension, type 2 diabetes, ischemic heart disease, anticoagulant use)
Presenting Symptoms	Acute left loin pain, fever, vomiting	Onset: sudden, severe flank pain radiating anteriorly; fever (101.5°F); associated nausea/vomiting
Physical Examination	Left costo-vertebral angle tenderness	Stable hemodynamics, mild dehydration, afebrile after admission
Laboratory Tests	Elevated serum creatinine (203 µmol/L); leukocytosis (WBC 15,600/µL); urinalysis: pyuria and microscopic hematuria	Coagulation profile prolonged due to anticoagulant therapy; Hb mildly reduced
Ultrasound KUB	Left hydronephrosis and hydroureteronephrosis	The right kidney showed fullness but no obvious stones
Non-contrast CT (NCCT)	Bilateral hyperdense lesions at the ureteropelvic junction, larger and more obstructive on the left side	Appearance suggestive of sloughed papillae; no calcified stones detected
Provisional Diagnosis	Bilateral renal papillary necrosis (RPN)	More prominent obstruction on the left side
Initial Management	Left percutaneous nephrostomy (PCN) for decompression	Attempted antegrade DJ stent malpositioned
Definitive Intervention	Flexible ureteroscopy	Loss of papillary impressions, sloughed papillae retrieved
Histopathology	Confirmed RPN	Necrotic tissue with papillary fragments
Follow-Up	PCN removed after nephrostogram showed patent ureter; patient asymptomatic at one month	Normal renal drainage, no hydronephrosis, stable renal function

Laboratory, hematologic, and histopathological findings

Serial laboratory parameters reflected the evolving course of infection, obstruction, and recovery. A marked leukocytosis was observed, rising from 6.7 ×10^9^/L on July 29 to a peak of 29.6 ×10^9^/L the next day, with >90% neutrophilic predominance, consistent with a severe acute bacterial process. Despite appropriate antibiotic therapy, leukocyte counts remained elevated for several days before gradually returning to normal levels with clinical improvement.

Hemoglobin levels gradually declined from approximately 10 g/dL to 7.7 g/dL during the acute phase, a pattern commonly observed in severe infection, inflammation, and obstructive uropathy. Although the red cell indices remained normocytic-normochromic - an appearance that does not fully exclude early or mixed-pattern iron deficiency - this profile is nonetheless typical of acute illness-related anemia and consistent with the patient’s septic and obstructive presentation. Platelet counts demonstrated reactive thrombocytosis during recovery, further supporting a resolving inflammatory state. Together, these hematologic trends aligned with the clinical course and reinforced the diagnosis of acute obstructive pathology secondary to RPN.

Histopathological evaluation of tissue retrieved from the left pelvicalyceal system confirmed the diagnosis of RPN. Microscopic examination revealed degeneration and fibrosis of renal parenchyma with eroded tubular epithelium, foci of calcification, and hemosiderin deposition, without any granulomatous or neoplastic changes. The 1.8 × 0.7 × 0.3 cm gray-tan tissue fragment represented sloughed necrotic papillae, consistent with ischemic papillary necrosis secondary to vascular compromise and prolonged anticoagulant therapy. A summary of findings is shown in Table 2.

**Table 2:** Summary of laboratory, hematologic, and histopathological findings.

Parameter	Normal Range	Findings/Values	Interpretation
WBC	4-10 × 10^9^/L	6.7 → 29.6 → 10.8	Marked neutrophilic leukocytosis with gradual recovery following treatment
Neutrophils (%)	40-80%	92% (peak)	Severe bacterial infection with systemic inflammatory response
Lymphocytes (%)	20-40%	3.8% (lowest)	Transient lymphopenia during acute infection
Hemoglobin	12-15 g/dL	9.9 → 7.7	Anemia of inflammation and hemodilution during sepsis
Platelets	150-400 × 10^9^/L	263 → 413	Reactive thrombocytosis during convalescence
Specimen (Histopathology)	-	Fibrotic tissue from the left pelvicalyceal system, measuring 1.8 × 0.7 × 0.3 cm	Sloughed necrotic papillae retrieved during ureteroscopy
Microscopic Features	-	Degenerated renal tissue with tubular erosion, fibrosis, calcification, and hemosiderin deposition	Consistent with ischemic renal papillary necrosis
Inflammatory/Neoplastic Changes	-	None detected	No granulomatous or malignant features
Final Diagnosis	-	Renal papillary necrosis with fibrosis of the pelvicalyceal system	Histopathologic confirmation of ischemic injury

Renal function and electrolyte profile

At presentation, the patient appeared clinically euvolemic, with no signs of peripheral edema, pulmonary congestion, or dehydration, helping contextualize the observed hyponatremia. Serum creatinine was markedly elevated at 306.9 µmol/L, with urea >22 mmol/L, consistent with acute renal dysfunction secondary to obstruction and sepsis. Electrolyte analysis demonstrated significant hyponatremia (Na^+^ 117-122 mmol/L) and hypochloremia (~90 mmol/L), while potassium levels remained within the normal range throughout. Daily urine output was initially low due to obstruction (<400-500 mL/day) but improved progressively following PCN on 02-08-2025, ultimately normalizing as drainage was restored. Renal function improved steadily thereafter, with creatinine declining to 190 µmol/L by 11-08-2025, and returning to ~150 µmol/L after definitive ureteroscopic clearance. Sodium and potassium values returned to the physiologic range, completing the patient’s biochemical recovery (Table 3).

**Table 3:** Serial kidney function test (KFT) parameters during hospital course. This table summarizes daily renal biochemical parameters, electrolyte status, urine output progression, and key procedural interventions from admission to recovery. Creatinine and urea values above the reference range are marked as "H," while sodium or chloride values below the reference range are marked "Low," and critical abnormalities are marked "Critically low." Urine output trends reflect the transition from oliguric obstructive AKI to post-decompression recovery. Potassium levels remained within normal physiological limits throughout hospitalization. Major interventions, including attempted DJ stent, PCN insertion, and flexible URS, are listed alongside corresponding clinical outcomes. PCN: percutaneous nephrostomy; DJ stent: double J stent; URS: ureteroscopy; AKI: acute kidney injury; UOP: urine output

Date	Creatinine (µmol/L) (62–106)	Sodium (mmol/L) (135–145)	Potassium (mmol/L) (3.5–5.1)	Chloride (mmol/L) (98–107)	Urea (mmol/L) (2.5–7.01)	Urine Output (mL/day)	Intervention/Procedure	Outcome/Notes (Including Imaging, Endoscopy, and Histopathology)
30-07-2025	306.9 (H)	122.7 (Low)	4.2	90.9 (Low)	22.25 (H)	<400	-	Obstructive AKI with oliguria; initial ultrasound showing hydronephrosis and echogenic debris
31-07-2025	284.7 (H)	122.2 (Low)	4.11	91.0 (Low)	22.53 (H)	<450	-	Persistent oliguric AKI; IV broad-spectrum antibiotics started
02-08-2025	291.9 (H)	117.4 (Critically low)	4.28	90.2 (Low)	21.21 (H)	≈350	Attempted antegrade DJ stent	Unsuccessful due to proximal ureteric obstruction (CT: obstructing stone, perinephric stranding)
02-08-2025 (22:07)	294.3 (H)	117.4 (Critically low)	4.25	90.5 (Low)	21.40 (H)	600	Left PCN insertion	Immediate drainage of turbid urine with debris; improved UOP
03-08-2025	293.8 (H)	118.0 (Critically low)	4.86	92.0 (Low)	21.20 (H)	900	-	Post-PCN improvement; hydronephrosis decompressing
03-08-2025 (11:27)	300.1 (H)	119.8 (Critically low)	4.50	92.6 (Low)	21.68 (H)	1100	-	Progressive renal recovery; electrolytes partially correcting
03-08-2025 (20:18)	300.1 (H)	121.7 (Low)	4.11	93.5 (Low)	22.22 (H)	1300	-	Continued improvement; stable hemodynamics
04-08-2025	296.4 (H)	122.0 (Low)	4.40	93.7 (Low)	22.24 (H)	1500	-	UOP progressively increasing
05-08-2025	284.6 (H)	125.9 (Low)	4.36	94.6 (Low)	20.10 (H)	1600	-	Renal function improving; electrolytes stabilizing
06-08-2025	252.7 (H)	126.3 (Low)	4.03	93.5 (Low)	19.06 (H)	1700	-	AKI resolving
06-08-2025 (04:44)	249.5 (H)	126.0 (Low)	4.06	93.8 (Low)	19.06 (H)	1700	-	Stable labs and UOP
07-08-2025	238.1 (H)	127.0 (Low)	4.35	93.6 (Low)	16.71 (H)	1800	-	Pre-operative assessment for URS
08-08-2025	216.0 (H)	130.2 (Low)	4.38	96.3 (Low)	17.52 (H)	1900	Flexible URS	Impacted stone cleared; sloughed renal papillae retrieved (suggestive of papillary necrosis)
09-08-2025	197.3 (H)	131.4 (Low)	4.28	98.1	15.78 (H)	2000	-	Post-URS: unobstructed drainage; improving renal function
10-08-2025	191.4 (H)	130.9 (Low)	4.66	98.4	14.07 (H)	2100	-	Continued renal recovery
11-08-2025	189.8 (H)	123.9 (Low)	4.31	94.0 (Low)	13.45 (H)	~2000	PCN removed	Renal function stabilized; electrolytes near-normal; recovery achieved
Histopathology	-	-	-	-	-	-	Tissue from URS	Sloughed necrotic renal papilla confirmed → Renal papillary necrosis (RPN) contributing to obstruction and ischemic injury

Radiological findings and interval imaging

**Figure 1:**
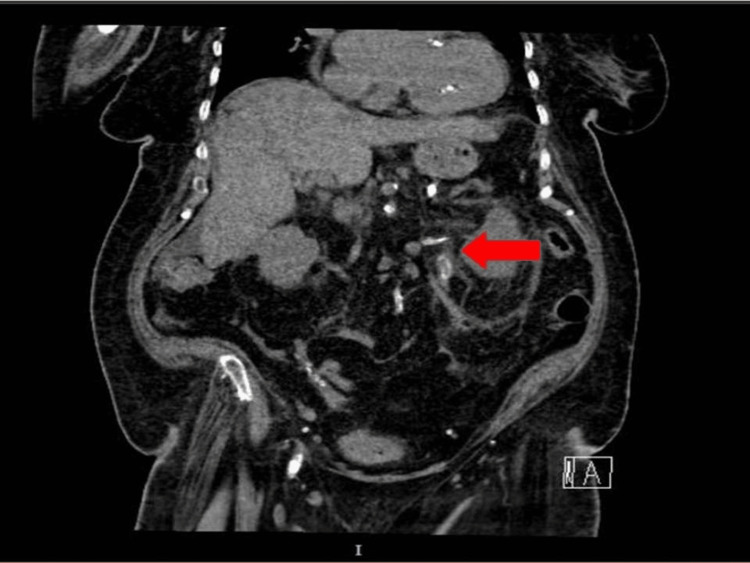
Non-contrast CT showing a calcified renal papilla in the left proximal ureter (arrow).

The initial non-contrast computed tomography (NCCT) of the kidneys, ureters, and bladder (CT KUB) demonstrated bilateral hyperdense lesions at the ureteropelvic junctions, with more pronounced findings on the left side, resulting in mild-to-moderate hydronephrosis. These lesions exhibited a density and morphology inconsistent with calcified calculi, strongly suggesting the presence of sloughed renal papillae. This was a key finding, as it raised suspicion for RPN, which was likely the cause of the patient’s obstructive uropathy (Figure 1). The configuration and attenuation pattern of the lesions were not typical of stones, reinforcing the diagnosis of RPN as the underlying etiology.

Subsequent fluoroscopic imaging after PCN insertion confirmed successful decompression of the left collecting system with immediate contrast drainage (Video 1). Flexible ureteroscopy (URS) was performed three weeks later, after the patient had achieved clear clinical stabilization, including normalization of inflammatory markers, return of serum creatinine to nadir under nephrostomy drainage, and a negative pre-operative urine culture. During URS, a substantial volume of sloughed and calcified necrotic papillary debris was encountered, and visualization was intermittently limited due to residual particulate matter-findings consistent with papillary necrosis rather than ongoing infection. The loop of the PCN was clearly visualized within the renal pelvis, and the obstructing papillary fragments were carefully retrieved using a Dormia basket (Coloplast Corp, Minneapolis, MN, USA). This staged approach allowed safe removal of the obstruction while minimizing postoperative risk, despite the inherently inflamed environment associated with papillary necrosis.

**Video 1:** Bilateral renal papillary necrosis in an elderly female, demonstrating successful percutaneous nephrostomy and ureteroscopy (URS).

**Figure 2:**
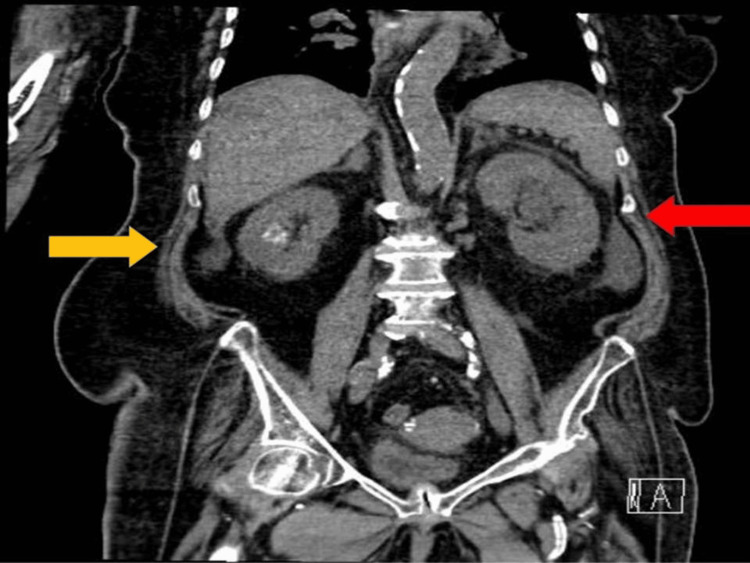
Non-contrast CT image showing bilateral hydronephrosis, more prominent on the left side. Yellow arrow: right kidney showing mild fullness; Red arrow: left kidney showing mild hydronephrosis on non-contrast CT.

During the PCN procedure, fluoroscopic guidance confirmed proper catheter placement into the left pelvicalyceal system with immediate contrast drainage, ensuring patency of the collecting system. This procedure effectively achieved rapid decompression, which led to a significant reduction in hydronephrosis, as well as clinical improvement in renal function and sepsis control. The immediate contrast drainage was pivotal in confirming the success of the intervention and the restoration of normal renal function (Figure 2).

Histopathological analysis

Microscopic examination of the retrieved tissue from the left pelvicalyceal system demonstrated degeneration and fibrosis of renal parenchyma with eroded tubular epithelium, foci of calcification, and hemosiderin deposition. Importantly, no granulomatous inflammation or neoplastic changes were observed. These findings confirmed RPN as the definitive diagnosis, correlating with the patient’s clinical and imaging profile. The tissue sample measured 1.8 × 0.7 × 0.3 cm, consistent with sloughed papillary material dislodged during obstruction. This histopathologic confirmation reinforced the ischemic etiology of papillary necrosis secondary to comorbid vascular compromise and prolonged anticoagulation.

During follow-up, serial CT scans demonstrated progressive resolution of hydronephrosis and perinephric stranding, stable nephrostomy and stent position, and improving inflammatory changes. The final contrast-enhanced nephrostogram showed free antegrade contrast flow from the renal pelvis into the bladder without obstruction, confirming complete restoration of urinary drainage.

**Figure 3:**
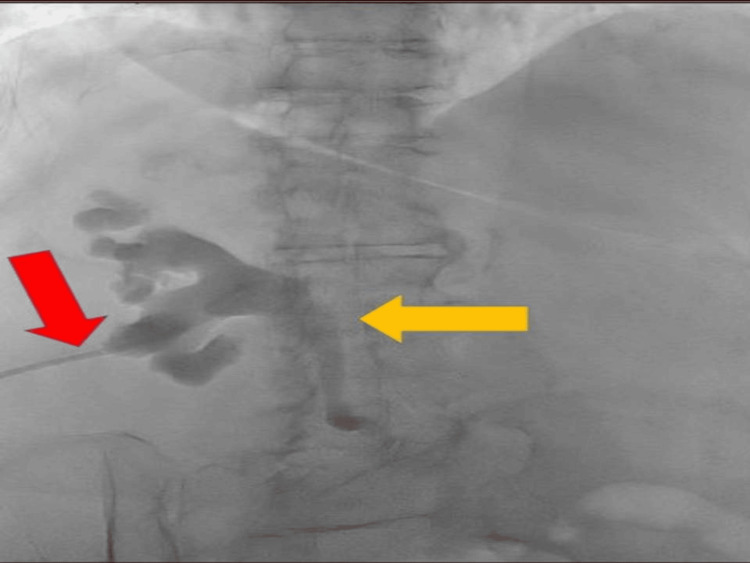
Pre-operative pyelogram prior to ureteroscopic clearance. The red arrow indicates the left-sided percutaneous nephrostomy (PCN) tube, and the yellow arrow highlights the dilated left ureter with distal obstruction.

The pre-operative antegrade pyelogram, obtained one week after stabilization and prior to definitive flexible URS, revealed persistent, irregular filling defects in the proximal ureter, representing sloughed papillary tissue and necrotic debris. Contrast study demonstrated delayed passage and poor peristaltic transit through the obstructed segment, with retained contrast in the dilated pelvicalyceal system, indicating continued partial obstruction despite prior decompression. Distal to the lesion, the ureter appeared smooth and contrast-filled, suggesting that the obstruction was localized and amenable to endoscopic management. These radiographic findings correlated precisely with the intraoperative URS observations of friable necrotic papillae within the ureteral lumen. During URS, these fragments were retrieved using a nitinol basket, and the affected area was gently irrigated until free antegrade flow was re-established. Post-procedure fluoroscopy confirmed complete clearance of debris and unobstructed drainage into the bladder, findings that were consistent with the final pyelogram and subsequent imaging follow-up (Figure 3).

**Figure 4:**
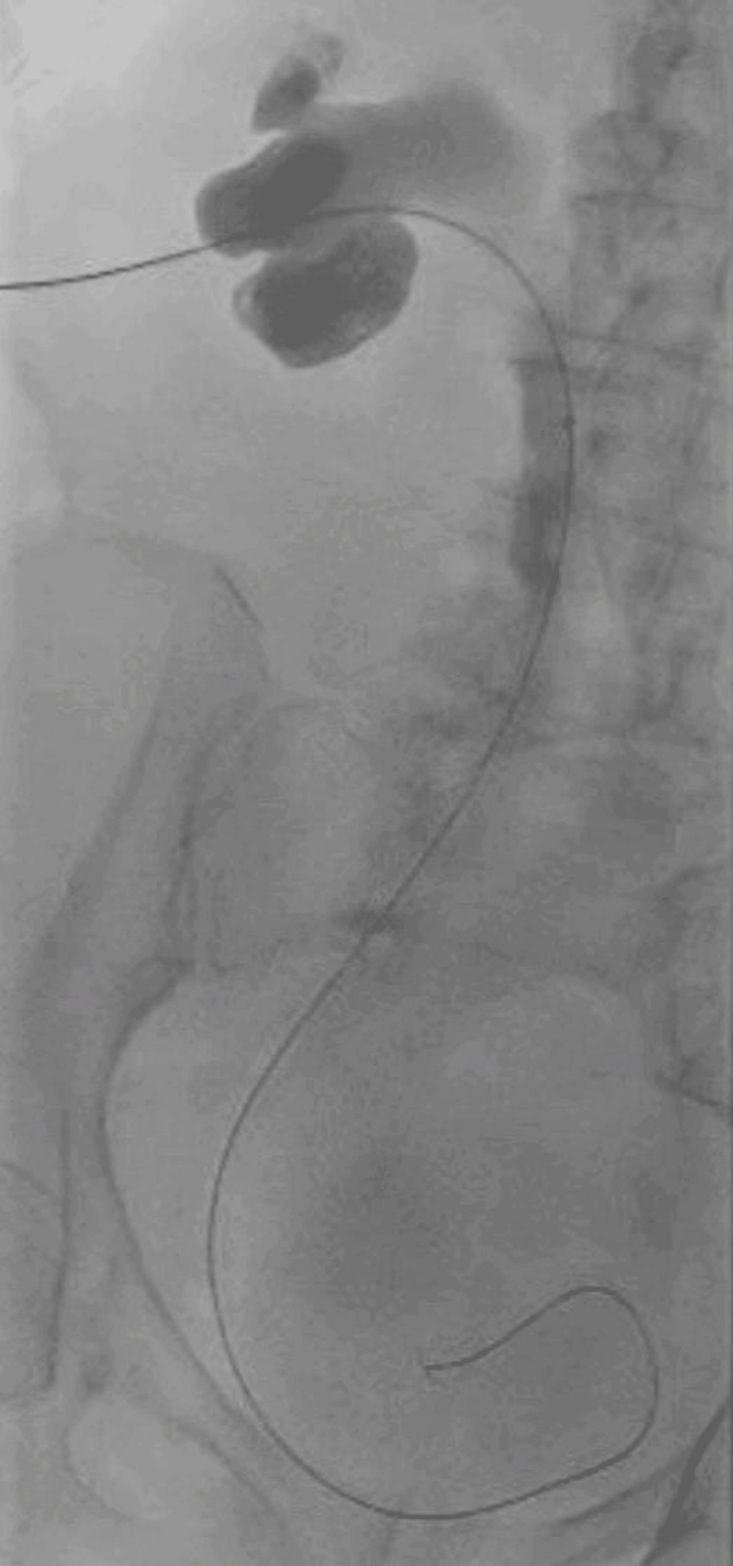
Antegrade pyelogram of the left kidney obtained during the attempted double-J (DJ) stent placement.

The antegrade pyelogram, performed at the time of attempted double-J (DJ) stent placement through the pre-existing nephrostomy tract, demonstrated opacification of the left pelvicalyceal system with an abrupt contrast cut-off at the proximal ureter, approximately 2-3 cm below the pelvi-ureteric junction. This finding indicated mechanical obstruction due to sloughed necrotic papillae, seen as irregular, non-calcified filling defects within the proximal ureteric lumen. The DJ stent tip failed to cross the obstruction and was visualized as malpositioned within the upper ureter, not reaching the urinary bladder. Despite multiple repositioning attempts under fluoroscopy, the obstructing tissue fragments prevented complete internal drainage, necessitating continued external diversion via the nephrostomy tube. The fluoroscopic study also showed mild residual hydronephrosis and slow clearance of contrast, consistent with partial decompression but ongoing proximal obstruction (Figure 4).

Subsequently, after clinical stabilization with PCN and partial recovery of renal function, definitive endoscopic intervention was performed. Flexible URS revealed extensive sloughed papillary debris within the proximal ureter, which was carefully retrieved using a nitinol Dormia basket under fluoroscopic guidance. Copious irrigation was used to clear residual fragments, and complete antegrade flow into the bladder was confirmed at the end of the procedure. This endoscopic clearance successfully resolved the obstruction, restored normal urine drainage, and allowed safe removal of the nephrostomy tube, achieving full decompression of the affected renal unit.

Follow-up and clinical outcome

The patient’s postoperative course was uneventful. Drain output through the nephrostomy tube progressively decreased, and a follow-up nephrostogram confirmed free antegrade passage of contrast into the urinary bladder without residual obstruction. The PCN was subsequently removed, and renal function normalized within two weeks. At one-month follow-up, she remained asymptomatic with no recurrence of flank pain, fever, or hydronephrosis on ultrasound.

Given the patient’s long-term anticoagulation for atrial fibrillation - a factor likely contributing to papillary ischemia and RPN - her anticoagulation regimen was carefully reassessed prior to discharge. Adjustments were made in consultation with cardiology to balance thromboembolic risk and the risk of further renal injury, and close follow-up was planned to monitor renal function, coagulation parameters, and hydration status (Table 4).

**Table 4:** Summary of key clinical, laboratory, and procedural parameters with trends. Data reflects temporal trends across hospitalization to highlight cause-and-effect between obstruction, interventions, and renal recovery. This summary consolidates laboratory, clinical, and procedural data, minimizing the potential for biased interpretation. PCN: percutaneous nephrostomy; URS: ureteroscopy; DOAC: direct oral anticoagulant; RPN: renal papillary necrosis

Parameter	Baseline/Initial	Peak/Maximum	Post-intervention/Recovery	Interpretation/Clinical Correlation
Serum Creatinine (µmol/L)	185	306.9	189.8	Acute kidney injury (AKI) due to obstruction; normalized post-PCN and URS
Serum Urea (mmol/L)	22	22.53	13.45	Correlates with obstructive AKI; improved with decompression
Sodium (mmol/L)	122.7	122.7	130.9	Hyponatremia during acute illness; corrected with fluid and obstruction relief
Potassium (mmol/L)	4.2	4.86	4.66	Within normal range throughout
Chloride (mmol/L)	90.9	93.8	98.4	Low during the acute phase; normalized with recovery
WBC (×10^9^/L)	6.7	29.6	10.8	Neutrophilic leukocytosis consistent with severe infection; normalized with therapy
Hemoglobin (g/dL)	9.9	7.7	11.2	Mild anemia due to inflammation and AKI; gradual recovery
Platelets (×10^9^/L)	263	413	350	Reactive thrombocytosis during convalescence
Urine Output (mL/day)	<400	1900	2000	Oliguria due to obstruction; improved after PCN and URS
Intervention	-	PCN insertion	Flexible URS	Staged intervention enabled stabilization and definitive clearance
Histopathology	-	Sloughed papillae	-	Confirms RPN; correlates with obstruction and ischemic injury
Anticoagulation	Long-term warfarin/DOAC	-	Adjusted perioperatively	Contributing factor to papillary ischemia; monitored during hospitalization

This case underscores the importance of early recognition, prompt decompression, and definitive endoscopic clearance in obstructive RPN, along with careful perioperative management of anticoagulation, to prevent irreversible renal impairment and minimize complications.

At presentation, the patient exhibited classic features of acute obstructive renal injury, including severe flank pain, fever, and reduced urine output. Laboratory evaluation revealed marked leukocytosis with neutrophilic predominance, mild anemia, and reactive thrombocytosis, reflecting a severe infectious and inflammatory process. Renal function tests demonstrated AKI with elevated serum creatinine and urea, accompanied by electrolyte disturbances, including hyponatremia and hypochloremia. Imaging studies revealed bilateral hydronephrosis, more pronounced on the left side, with hyperdense lesions suggestive of sloughed papillae causing obstruction. Initial decompression was achieved via PCN, followed by definitive flexible ureteroscopic retrieval of necrotic papillary fragments. Histopathology confirmed ischemic RPN without evidence of malignancy or granulomatous disease. Serial monitoring showed progressive improvement in renal function, normalization of inflammatory markers, and restoration of unobstructed urinary drainage, correlating closely with the timing of interventions and clinical recovery.

Ethical considerations

Written informed consent was obtained from the patient and her family for all procedures, for publication of this case, and for inclusion of relevant clinical images. The study adhered to institutional ethical standards and the principles outlined in the Declaration of Helsinki.

## Discussion

RPN classically occurs in patients with multiple predisposing factors and can present with fever, flank pain, and obstructive uropathy when sloughed papillae migrate into the collecting system. Historic clinicopathologic series reports that RPN is often bilateral and frequently presents with chills, fever, and flank pain-features that closely matched our patient’s presentation [2,5,6]. While conditions such as diabetes, analgesic abuse, and severe infection remain leading etiologies, systemic factors such as vascular disease or anticoagulation may also predispose to papillary ischemia [1,7]. In our case, long-term anticoagulation for atrial fibrillation may have contributed to papillary ischemia. Anticoagulation therapy can promote microvascular bleeding within the metabolically vulnerable renal papillae, resulting in local ischemia, tubular obstruction, necrosis, and sloughing of papillary tissue. This process can be exacerbated by comorbid vascular disease or systemic infection, creating a “two-hit” scenario that increases the risk of obstructive AKI. Perioperative management of anticoagulation added complexity to her care, consistent with prior observations [7]. No autoimmune workup was performed as the clinical presentation, risk profile, imaging, and histopathology strongly supported an ischemic/obstructive etiology.

Imaging findings in RPN are variable and may mimic stones or tumors when sloughed papillae create filling defects or hyperdense intraluminal material on CT. Several authors emphasize that NCCT may reveal hyperdense filling defects at the pelvi-ureteric junction or within the ureter, but radiologic diagnosis can be challenging without direct visualization [2,8]. Our NCCT demonstrated bilateral hyperdense lesions, initially mistaken for calculi, but ureteroscopic evaluation later confirmed sloughed papillae as the source of obstruction. This highlights the importance of correlating imaging with clinical suspicion, as emphasized in prior literature [4,8].

Management strategies range from conservative medical care (antibiotics, withdrawal of offending drugs) to interventional procedures, including urinary decompression and endoscopic or percutaneous removal of necrotic papillae. Case reports document successful clearance of sloughed papillae with rigid or flexible URS, and in certain scenarios, percutaneous retrieval has been effective in transplant and non-transplant kidneys [2,9]. In our patient, a flexible URS was performed after initial stabilization with PCN. Although a significant amount of debris was visualized and visualization was occasionally suboptimal due to residual inflammation, careful endoscopic clearance was safely achieved.

It should be noted that, depending on the size and mobility of sloughed papillae, spontaneous passage with the urinary stream can occur. Therefore, immediate endoscopic intervention may not be necessary in every patient. Following successful decompression and clinical stabilization, a period of observation with repeat imaging may allow for resolution of infection and assessment of residual obstruction, potentially preventing unnecessary procedures. In cases with persistent obstruction or biochemical derangements, timely ureteroscopic retrieval remains indicated to prevent ongoing renal injury or recurrent infection. This approach underscores the importance of individualized decision-making, balancing early obstruction relief with adequate infection control [2,9].

The role of PCN prior to definitive URS is particularly noteworthy. Studies demonstrate that PCN provides immediate drainage, aids in controlling infection, and improves the success and safety of subsequent endourological procedures [10]. In our patient, PCN insertion resulted in prompt clinical improvement and allowed for safer ureteroscopic intervention under optimized conditions.

Outcomes in published reports vary according to underlying etiology and timeliness of intervention. Severe infection-related RPN carries a higher risk of morbidity and mortality [1,5], whereas cases with obstructive fragments managed promptly with decompression and endoscopic clearance often demonstrate good renal recovery and minimal long-term sequelae [2,6,9]. Our patient’s full recovery at one month is consistent with these favorable outcomes.

Strengths and limitations

A strength of this report is the stepwise, multidisciplinary management (critical care, nephrology/urology, careful anticoagulation handling) that produced a good outcome. Limitations include that ours is a single case and that follow-up was short (one month), whereas some series document longer follow-up and note recurrence or late complications in a minority of patients. Imaging alone was non-diagnostic and required ureteroscopic confirmation and histopathology; this reiterates literature recommendations that endoscopic assessment is often required for definitive diagnosis when radiology is equivocal.

Clinical implications

This case emphasizes (1) RPN should be considered when CT shows unexplained hyperdense intraluminal material in patients with risk factors; (2) anticoagulation may contribute to renal papillary ischemia and should be carefully managed perioperatively; (3) immediate decompression (PCN) is critical in septic or obstructed patients and facilitates safer definitive endoscopic clearance; and (4) individualized timing of endoscopic retrieval should consider spontaneous passage, infection resolution, and residual obstruction, balancing patient safety and procedural necessity. Future case series with longer follow-up would help clarify recurrence risk and optimal surveillance strategies.

## Conclusions

This case illustrates the diagnostic and therapeutic challenges of RPN in elderly patients with multiple comorbidities. The initial presentation closely resembled obstructive uropathy due to calculi; however, imaging findings of bilateral hyperdense lesions and subsequent endoscopic confirmation of sloughed papillae established RPN as the underlying cause. Early recognition of this differential diagnosis is vital, as delayed intervention can result in irreversible renal injury, sepsis, or mortality.

Successful management of this patient was achieved through a stepwise, minimally invasive approach - initial PCN for urgent decompression, followed by definitive flexible ureteroscopic clearance of necrotic papillae. Histopathological confirmation validated the diagnosis, and the patient achieved complete clinical and functional recovery at follow-up. This case emphasizes that timely intervention, guided by multidisciplinary coordination, can yield excellent outcomes even in high-risk elderly patients with complex systemic conditions.

## References

[1] Gaudji GR, Bida M, Conradie M, Damane BP, Bester MJ (2022). Renal papillary necrosis (RPN) in an African population: disease patterns, relevant pathways, and management. Biomedicines.

[2] Pan HH, Luo YJ, Zhu QG, Ye LF (2022). Renal papillary necrosis with urinary tract obstruction: a case report. World J Clin Cases.

[3] Yaxley J, Yaxley W (2023). Obstructive uropathy - acute and chronic medical management. World J Nephrol.

[4] Jain V, Sureka B, Bansal K, Arora A (2015). Obstructive uropathy: is it always urolithiasis?. Indian J Nephrol.

[5] Eknoyan G, Qunibi WY, Grissom RT, Tuma SN, Ayus JC (1982). Renal papillary necrosis: an update. Medicine (Baltimore).

[6] Glusman ZA, Sample KJ, Landau KS, Vigo RB (2020). Renal papillary necrosis following mesenteric artery stenting. Cureus.

[7] Kalaitzidis RG, Duni A, Liapis G, Balafa O, Xiromeriti S, Rapsomanikis PK, Elisaf MS (2017). Anticoagulant-related nephropathy: a case report and review of the literature of an increasingly recognized entity. Int Urol Nephrol.

[8] Jung DC, Kim SH, Jung SI, Hwang SI, Kim SH (2006). Renal papillary necrosis: review and comparison of findings at multi-detector row CT and intravenous urography. Radiographics.

[9] Kamath S, Moody MP, Hammonds JC, Wells IP (2005). Papillary necrosis causing hydronephrosis in renal allograft treated by percutaneous retrieval of sloughed papilla. Br J Radiol.

[10] Kwon SY, Kim BS, Kim HT, Park YK (2013). Efficacy of percutaneous nephrostomy during flexible ureteroscopy for renal stone management. Korean J Urol.

